# Homology-based method for detecting regions of interest in colonic digital images

**DOI:** 10.1186/s13000-015-0244-x

**Published:** 2015-04-24

**Authors:** Kazuaki Nakane, Akihiro Takiyama, Seiji Mori, Nariaki Matsuura

**Affiliations:** Department of Molecular Pathology, Osaka University Graduate School of Medicine and Health Science, 1-7 Yamadaoka, Suita, Osaka 565-0871 Japan; Department of Cancer Pathology, Hokkaido University Graduate School of Medicine, Nishi 7 kita 15 Kita ward, Sapporo, Hokkaido 060-8638 Japan

**Keywords:** Pathology, Colon cancer, Computer-assisted diagnosis, *The Betti numbers*, Histology

## Abstract

**Background:**

A region of interest (ROI) is a part of tissue that contains important information for diagnosis. To use many image analysis methods efficiently, a technique that would allow for ROI identification is required. For the colon, ROIs are characterized by areas of stronger color intensity of hematoxylin. Since malignant tumors grow in the innermost layer, most ROIs will be located in the colonic mucosa and will be an accumulation of tumor cells and/or integrated cells with distorted architecture.

**Methods:**

Using homology theory, our group proposed a method to estimate the contact degree of elements in a unit area of tissue. Homology is a concept that is used in many branches of algebra and topology, and it can quantify the contact degree. Due to the lack of contact inhibition of cancer cells, an area with unusual contact degree is expected to be a potential ROI.

**Results:**

The current work verifies the accuracy of this method against the results of pathological diagnosis, based on 1825 colonic images provided by the Osaka Medical Center for Cancer and Cardiovascular Diseases. Although we have many false positives and there is a possibility of missing undifferentiated types of cancer, this system is very effective for detecting ROIs.

**Conclusions:**

The mathematical system proposed by our group successfully detects ROIs and is a potentially useful tool for differentiating tumor areas in microscopic examination very quickly. Because we use only the information from low-power field images, there is room for further improvement. This system could be used to screen for not only colon cancer but other cancers as well. More sophisticated and more efficient automated pathological diagnosis systems can be developed by integrating various techniques available today.

**Virtual Slide:**

The virtual slide(s) for this article can be found here: http://www.diagnosticpathology.diagnomx.eu/vs/7129390011429407.

## Background

Building a reliable computer-assisted pathological diagnosis system will help reduce the burden on pathologists. Various methods have been proposed, but cancer tissue is difficult to recognize because of its complex morphology. Moreover, with the development of virtual slides, biopsy samples can be easily digitized. The amount of data to be processed has increased significantly, but current systems for processing enormous databases are expensive and obtaining numerical results is time-consuming.

A region of interest (ROI) is a part of tissue that contains important information for diagnosis. Detailed and efficient numerical results could be obtained if there was a way to combine established image analysis methods to identify ROIs from a whole-slide image quickly. In a typical case, tumor cells have hyperchromatic nuclei that include condensation of heterochromatin, which can be stained with hematoxylin [1]. Furthermore, malignant tumors grow in the innermost layer; therefore, most ROIs will be located in the colonic mucosa and will be an accumulation of tumor cells and/or integrated cells with distorted architecture. Hence, we suppose that ROIs are characterized by areas of stronger color intensity of hematoxylin.

**Figure 1:**
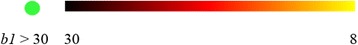
The colored dot is placed at the left edge of a segment, and the color represents the value of the Betti numbers, as shown by the color bar.

**Figure 2:**
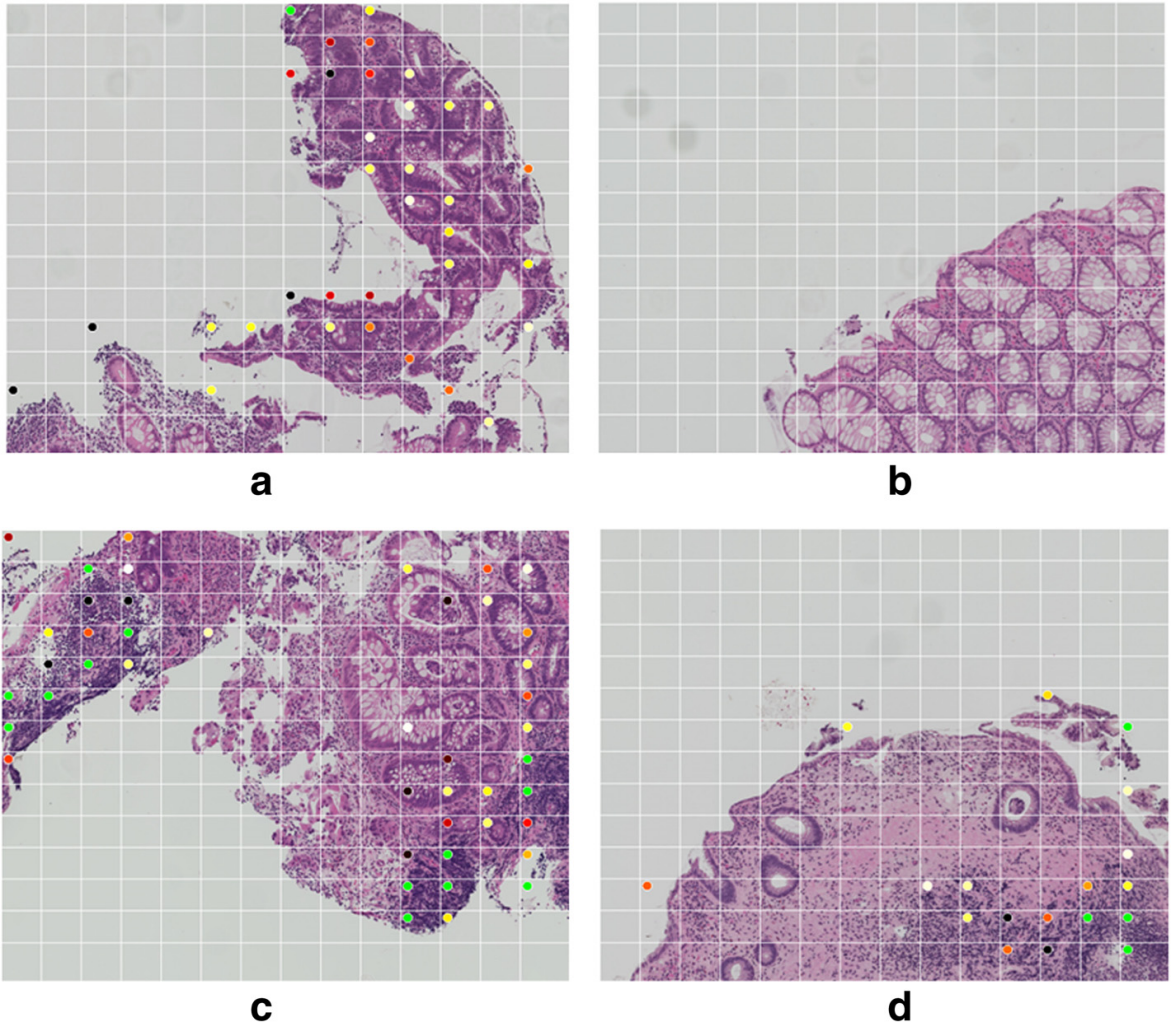
Numerical results of our method. **(a)** True Positive: Atypical glands. **(b)** True Negative: Mild colitis, non-neoplastic mucosa. **(c)** False Negative: Individual cells and small collection of tumor cells are not detected. On the other hand, lymphoplasmacytic infiltrate causes inappropriate detection. **(d)** False Positive: Concentration of lymphocytes. The segments where the dot is placed include the ROI.

Recently, our group proposed a simple mathematical model for the identification of tumor areas within normal tissue utilizing the changes in *the Betti numbers* in tumorigenesis [2]. Using the concept of *the Betti numbers* (homology), it is possible to evaluate quantitatively the contact degree between two points in a figure (see Figure 1, [2]). The concept of homology is a modern mathematical tool [3-5], and largely unknown. While expert knowledge of mathematics is required to fully comprehend homology, in two-dimensional cases, such as image analysis, use of homology is quite simple. In this case, *the Betti numbers* consist of two numbers: *b0* (*the 0-dimensional Betti number*), which is the number of isolated solid components (a cell or cell nucleus), and *b1* (*the 1-dimensional Betti number*), which is the number of windows in the fenestrated area. These areas are created by incomplete fusion of neighboring isolated solid components. In this paper, we introduce our numerical results and verify the effectiveness of the method for detecting ROIs. Here, *b1* is used for simplicity as the index.

### Concept of our algorithm

Lesions can be considered areas with “different contact degrees”. Since homology is a mathematical tool to quantify “the contact degree”, it is possible to apply this idea to detect a lesion area in a digital image.

### Advantage of the proposed method, 1: *Topological invariant*

Because tissue composition is nonuniform, applying pattern recognition methods is extremely difficult. There is a concept in homology theory called *the topological invariant*, and it represents a quantity that is unchangeable by continuous transformation. *The Betti numbers* are topological invariants. By applying this concept, the numerical results in the proposed method remain uninfluenced by slight differences in shape.

### Advantage of the proposed method, 2: Average in the unit area

Localized differences are inevitable in living tissue. The proposed method is able to evaluate the calculation results in each unit area; therefore, the results are not affected by this localized difference.

There are no specific criteria for defining the size of a unit area. It is believed that the unit area size will depend on the characteristics of a given tissue.

## Material

Colonic specimens were provided by the Osaka Medical Center for Cancer and Cardiovascular Diseases. They included biopsy, endoscopic mucosal resection and surgical specimens. Data were gathered for internal quality control on a routine basis and all patients gave informed consent for data collection. This study was approved by the institutional review board (IRB- the Osaka Medical Center for Cancer and Cardiovascular Diseases). They were stained with hematoxylin and eosin and scanned by a Nano-Zoomer 2 (Hamamatsu Photonics K. K.). The WSIs (whole-slide images) obtained from this virtual slide were divided into several bitmap images. These colonic images (magnification: 100, total images: 1825) were processed using a conventional laptop computer (Dell Vostro, Intel Core i7-3632QM, 2.20 GHz, 4.00 GB).

## Methods

The binarize parameter is determined automatically from the RGB (red-green-blue) information for each image. Because binarized images are mathematical objects, the *b1* value can be calculated. CHomP [6] was used to obtain numerical results.

Each image was then decomposed into 14 × 14 segments, and *b1* for each segment was obtained. A colored dot is placed at the left edge of a segment (see Figure 2); the color represents the value of *b1* (see Figure 1). The segments with a green dot are segments where the value of *b1* is very high (i.e., *b1* > 30).

Using a laptop computer, the process takes approximately 2.0–3.0 seconds per image. Since the system that was used has not been parallelized, the computations can be faster.

Generally, the tissues (structures) are constructed by the contact between the components. Because this method calculates the contact degree of the tissues (structures), we can apply it to many fields (cf. [7-13]).

## Results

The results of our numerical calculations are shown in Table 1. Figure 2 shows typical examples of each sample. We can see that there are a considerable number of false positives. This is because the algorithm is measuring the extent of accumulation in the tissue composition, so even non-cancerous cells are detected. As shown in Figure 2(c), tissues with an inconsistent cellular architecture are difficult to measure using this technique.

**Table 1:** **Contingency table as the cancer detector**

	**Cancer**	**Benign**
Positive	1284	422
Negative	1	118
Sensitivity	99.9	
Specificity	21.9	
False positive	78.1	
False negative	0.1	

Pathologists are typically able to identify the cancerous region immediately. However, as a screening system, automatic detection of the region that contains the data required for pathological diagnosis (i.e., the ROI) would be useful. Thus, we herein confirm whether this system is effective in detecting the ROI.

Although the ROI is itself the subject of debate in the field of oncology, we have proposed ROI classifications, as shown in Table 2. Specifically, we propose that a ROI might contain the following: (1) mild atypia; (2) mucosal inflammation; (3) hyperplastic polyps; (4) inflammatory cells; (5) regenerative change; (6) necrosis; (7) lymphoid follicles; (8) lymphocyte aggregation. It is often difficult to differentiate regenerative changes from neoplastic atypia, so we consider that the above components contain the information needed for pathological diagnosis. For the purpose of this study, we have therefore selected these components to represent the ROI. Moreover, a single image may contain multiple types of ROI. In this case, the classification is made based on the ROI having the largest size. The contingency table as the ROI detector is shown in the Table 3.

**Table 2:** **An itemized list of false positives**

**ROI**		**Non-ROI**	
Mild atypia	156	Cross sections of inclined glands	73
Mucosal inflammation	99	Numerical artifact	16
Hyperplastic polyps	34	Specimen artifact	2
Inflammatory cells	16		
Regenerative change	12		
Necrosis	6		
Lymphoid follicles	4		
Lymphocyte aggregation	4

**Table 3:** **Contingency table as the ROI detector**

	**ROI**	**Benign**
Positive	1615	91
Negative	1	118
Sensitivity	99.9	
Specificity	56.5	
False positive	43.5	
False negative	0.1	

**Figure 3:**
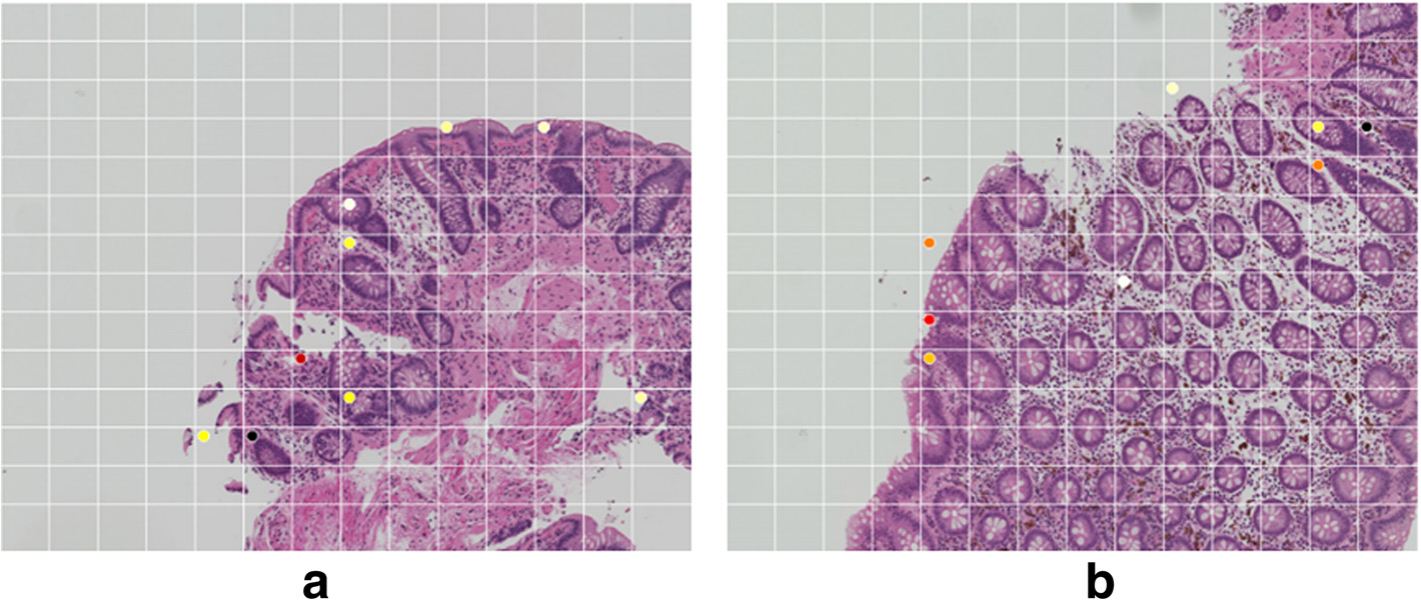
The non-ROI false-positive example (normal tissues). **(a)** False Positive: Tubular adenoma with mild atypia. The marked segments include cross sections of inclined glands. **(b)** False Positive: The marked segments include cross sections of inclined glands.

**Figure 4:**
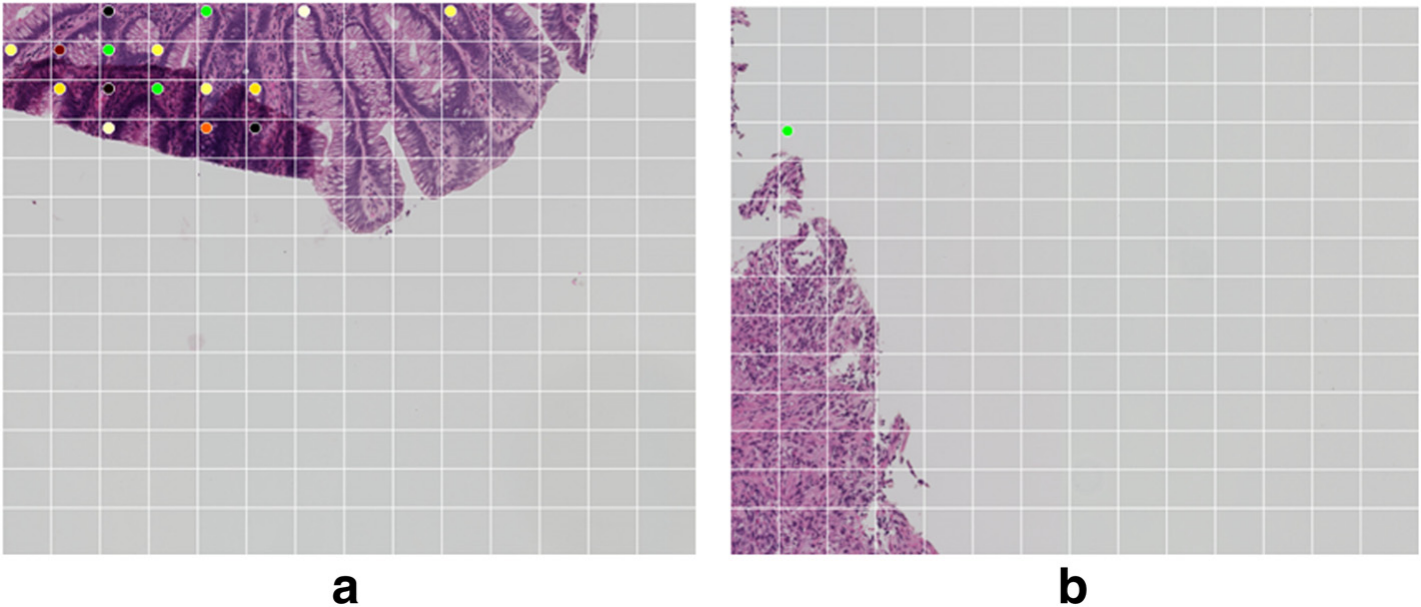
The non-ROI false-positive example (artifacts). **(a)** Folded sample: In the folded area, because the sample is overlapped, the homology values are high. **(b)** Numerical artifact: For normalization, we divide *the Betti numbers* in a ratio of non-blank area. If the non-blank ration is very small, the normalized result is very high.

The samples in Figure 3 show cross sections of inclined glands. The microscopic images certainly indicate a high level of accumulation. The cross sections of inclined glands are unrelated to the lesion, so they must be regarded as a non-ROI. The images in Figure 4 show the folded samples and the numerical artifact. In the folded area, because the sample is overlapped, the homology values are high. For normalization, we divide *the Betti numbers* in a ratio of non-blank area. If the non-blank ration is very small, the normalized result is very high. Although we have many false positives and there is a possibility of missing undifferentiated types of cancer, this system is very effective for detecting ROIs.

## Discussion

There are several approaches in the literature for automatic detection of colon cancer in digital tissue images. Altunbay et al. introduced four different approaches, namely, morphological, intensity-based, textural, and structural approaches [14]. The morphological approaches use classical geometrical properties such as size, area, and perimeter in tissue quantification. However, there is a difficult segmentation problem with these approaches because of the complexity of tissue images. The intensity-based approaches use gray level or color intensities of pixels, and calculate a histogram and define an average, standard deviation, entropy, and so on. However, similar color distributions of hematoxylin–eosin stain make these approaches difficult. The textural approaches use texture on pixels, and so are easily affected by artificial noise. Rathore et al. categorized these approaches from a different perspective into three techniques, namely, texture analysis, object-oriented texture analysis, and spectral analysis [15]. Their assessment revealed that none of the techniques is perfect.

In this paper, we introduced a completely different approach, that is, a homology method, using topological invariants—*the Betti numbers*. Our method accurately detects atypical epithelia regarded as carcinoma and high-grade adenomas that have a high nuclear-cytoplasmic ratio. The microscopic images of these tissues show increased contact between tumor cell nuclei due to their enlargement and pseudo-stratification. Consequently, *the Betti numbers* of these tissues are increased.

The epithelial tissues showing false positives classified as ROI all share a common trait in the form of enlarged, elongated nuclei and, occasionally, increased chromatin. In the microscopic images, the nuclei of these tissues all exhibit increased contact. That is why setting an algorithm to detect the ROI by computer results in these tissues being detected as positives. Put differently, our technique correctly detected atypical epithelia as a ROI candidate.

Conversely, neoplastic atypia for which the nuclear-cytoplasmic ratio was not particularly highly seen in low-grade adenoma, non-neoplastic, regenerative atypia, and proliferative zone were detected as false positives. A new algorithm needs to be added to identify these components.

To reduce the number of non-ROI, it is necessary to distinguish the cross sections of inclined glands. The pathologists typically make a differential assessment while subconsciously considering the global tissue structure, and will therefore assess these components as negative. The question of how to integrate this thinking into an algorithm is a matter that requires further deliberation. Furthermore, establishing a method to distinguish between neoplastic atypia and non-neoplastic atypia (regenerative atypia and proliferative zone) may lead to the development of a more practical tool. It is essential to discern whether the increase in contact was characterized by a constant nuclear polarity, in other words the same alignment, or by nuclei with disordered polarity and irregular alignment.

We obtained our results using only low-power microscopy. If conglomerations appear in the chromatin of tumor cells, topological invariants would be changed in the nucleic region. Using our method in combination with high-power microscopy would improve specificity. For detecting the area of undifferentiated carcinoma, we should use a specialized pattern recognition technology. Although we have assessed only colonic images, our system could be used to screen for not only colon cancer but other cancers as well. In addition, we have not identified the value of the homology with the convalescence. Because our method can be used to index cancer tissue, we can link the results with other pathological data. This will be done in a future study.

## Conclusion

The proposed mathematical system successfully detects ROIs and is a potentially useful tool for differentiating tumor areas in microscopic examination. By combining this newly introduced method and other approaches, we expect further improvements in the automatic detection of colon cancer.
